# Evaluation of groundwater resources in Wadi Qena, Egypt: a geophysical and hydrogeochemical perspective

**DOI:** 10.1038/s41598-025-29240-7

**Published:** 2025-12-12

**Authors:** Mohamed Khalifa, Mahmoud S. Sharkawy, Mahmoud Mohamaden, Ayman Taha, Ahmed Abdel Moneim, Sameh Zaki, Ahmed M. Masoud

**Affiliations:** 1 National Research Institute of Astronomy and Geophysics (NRIAG), Helwan, Cairo, 11722 Egypt; 2 Department of Petroleum Geology, Faculty of Petroleum and Mining Sciences, Matrouh University, Marsa Matrouh, Matrouh 51511 Egypt; 3 National Institute of Oceanography and Fisheries (NIOF), Alexandria, Egypt; 4 Geology Department, Faculty of Science, Sohag University, Sohag, 82524 Egypt; 5 The Egyptian Mineral Resources Authority, Cairo, 11517 Egypt

**Keywords:** Geophysics, Groundwater, Wadi Qena, TDEM, Magnetic survey, Nubian sandstone aquifer systems, Hydrogeology, Hydrochemistry, Arid regions

## Abstract

An integrated hydro-geophysical and hydrochemical investigation was conducted to delineate aquifer geometry, assess groundwater potential, and evaluate water quality in the southern part of Wadi Qena, Eastern Desert, Egypt. Eighteen time-domain electromagnetic (TDEM) soundings, ground magnetic profiles, pumping-test data, and six groundwater chemical analyses were jointly interpreted. The integrated datasets reveal five geo-electrical layers and identify two main aquifer systems: a shallow Quaternary aquifer (50–300 m depth; 4.9–86 Ω m) and a deeper Nubian Sandstone aquifer (300–650 m depth; 4.7–17.6 Ω m). Magnetic modeling delineates a variable basement surface (350–850 m) that controls aquifer thickness and the spatial distribution of transmissive zones. Areas of deep basement lows coincide with high-transmissivity wells (655–1170 m^2^/day) and low resistivity, indicating thick, well-connected sandstone bodies. Hydrochemical data (TDS: 1447–1607 mg/L; Na–Cl facies) indicate increasing salinity toward the northwest, consistent with upward leakage along magnetic lineaments and the dissolution of salt-bearing formations. The integrated interpretation demonstrates that combining TDEM, magnetic, and geochemical approaches provides a robust framework for identifying productive aquifers, understanding salinity sources, and optimizing groundwater development in arid terrains.

## Introduction

Water resources in arid and semi-arid regions are facing increasing pressure due to climate change, population growth, and the expansion of agricultural and industrial activities. Egypt, which has limited freshwater sources and relies heavily on the Nile River, has placed increasing emphasis on exploring other sources of water, especially groundwater.

Wadi Qena, a desert area located in the Eastern Desert of Egypt, represents one of the promising areas for agricultural development due to its geological and geomorphological characteristics, where groundwater plays a crucial role in sustaining both human livelihoods and ecosystems^1–3^.

However, effective management and utilization of groundwater resources require a comprehensive understanding of subsurface characteristics and hydrogeological conditions. Therefore, there is an urgent need to conduct detailed investigations using comprehensive geophysical and hydrogeological methods to investigate and assess the groundwater resources in Wadi Qena.

Electromagnetic techniques have become powerful non-invasive approaches for exploring subsurface groundwater systems, offering essential information on aquifer characteristics, flow dynamics, and structural influences^4–7^.

Despite its importance, the assessment of groundwater potential and subsurface characteristics in the Wadi Qena area remains limited and restricted to some research that deals with groundwater exploration using VES and aeromagnetic geophysical methods, as well as a few hydrogeological studies^8–11^. Moreover, many studies have addressed the integration of different geophysical methods and hydrochemical aspects to find groundwater potential in various areas of Egypt^12–16^.

The goal of this study is to evaluate the groundwater potentiality and subsurface characteristics in the Wadi Qena area of Egypt, utilizing advanced hydro-geophysical methods and techniques. By integrating various geophysical tools, including time-domain electromagnetic (TDEM) and magnetic surveys, this research aims to delineate aquifer geometry and characterize subsurface structures. Moreover, a pumping test helps determine aquifer parameters, which are useful in understanding aquifer productivity and optimal pumping operating rates. Hydrochemical data analysis also helps in detecting groundwater quality, which determines the sustainability and development potential of the region. Such insights are essential for sustainable groundwater management, informed decision-making, and the development of effective conservation strategies in arid regions like Wadi Qena.

### Study area description

**Fig. 1:**
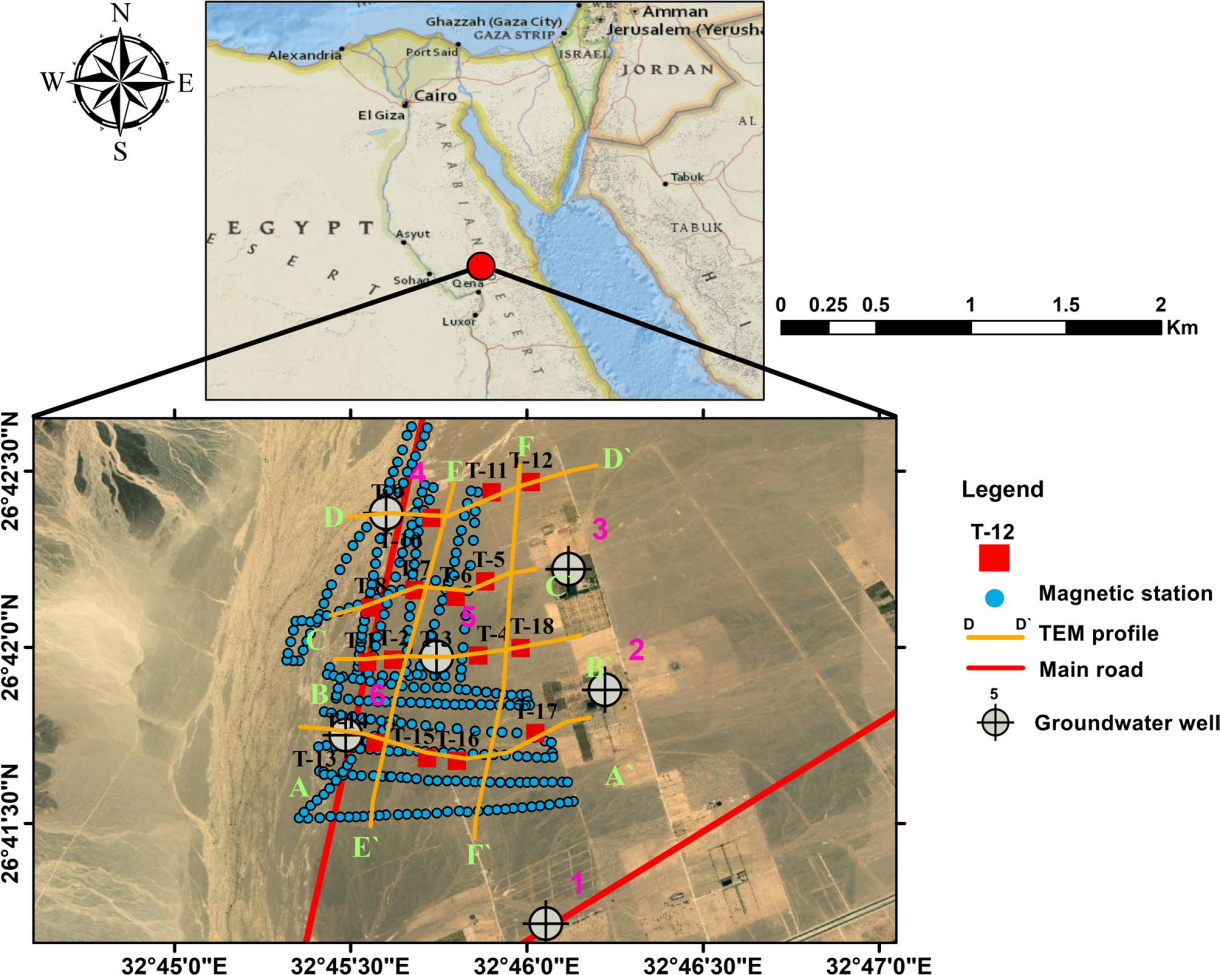
Location map of the study area.

The study area, located in the southern part of Wadi Qena, is bounded by longitudes 26°41ʹ01ʺ and 26°42ʹ35ʺ E and latitudes 32°45ʹ20ʺ N and 32°46ʹ20ʺ N (Fig. 1). The region is characterized by a dry environment with limited precipitation and high rates of evaporation. Its position in an arid desert defines it with scorching summers and frigid winters.

### Geologic setting

**Fig. 2:**
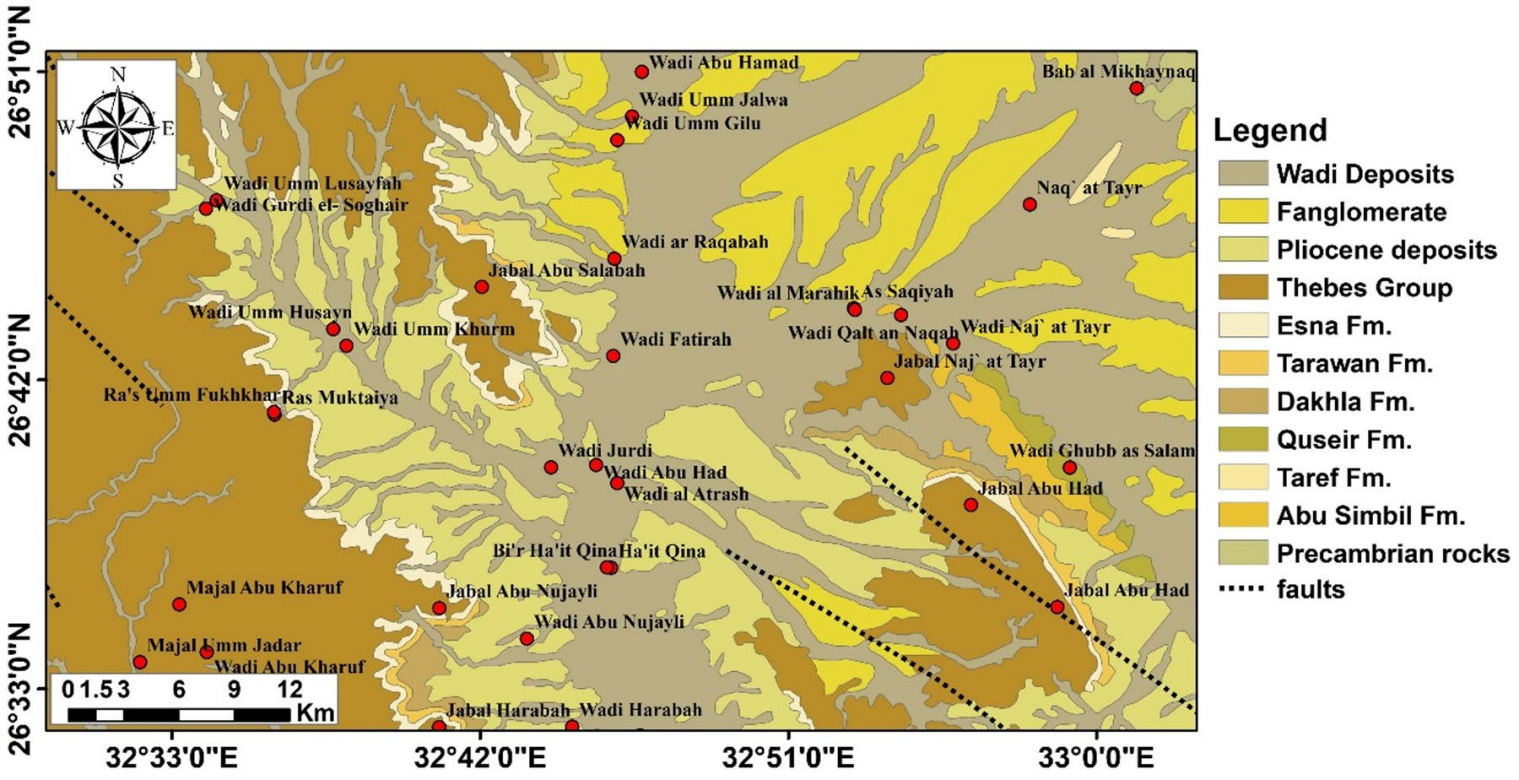
Geologic map of the study area^22^.

The Wadi Qena area of Egypt is situated within the Eastern Desert region, characterized by its sedimentary cover that overlies the complex Precambrian component (Fig. 2). The region is predominantly composed of Precambrian basement rocks, including granites, gneisses, and schists, which form the underlying bedrock representing the stable continental crust of the Arabian-Nubian Shield, which underwent extensive tectonic activity during the Pan-African orogeny around 600 million years ago^17^. Overlying the Precambrian basement rocks are sedimentary formations of the Mesozoic age, comprising fluvial sandstone (Nubian Sandstone) of Abu Simbil and Taref Formations and shales of Quseir Formation (Fig. 2). These sedimentary deposits were primarily formed during the Jurassic and Cretaceous periods when the region experienced marine transgressions and regressions^18^. The Nubian Sandstone, comprising predominantly porous and permeable sandstone layers, represents a significant aquifer system in the area^19^. Paleogene deposits are predominant in the Wadi Qena area, comprising the Dakhla Shale, Tarwan Chalk, Esna Shale, and Thebes Limestone formations. The Quaternary Pleistocene sand represents the upper saturated layer, while the Pliocene clay signifies the aquitard layer within the aquifer. Fanglomerates and wadi deposits are both recent deposits in the study area. The tectonic history of the Eastern Desert has been shaped by multiple phases of rifting and faulting, including the opening of the Red Sea rift during the Oligocene to Miocene epochs^20^. This tectonic activity has led to the formation of structural features, such as faults, folds, and grabens, which in turn influence the distribution and movement of groundwater within the subsurface^21^.

## Materials and methods

### Electromagnetic survey (TDEM)

The Transient Electromagnetic (TDEM) method was applied to investigate subsurface resistivity variations and to provide detailed information on groundwater-bearing formations. A total of 18 TDEM soundings were acquired in September 2021 using the AIE-2 system with a square loop measuring 200 × 200 m^2^ in a single-loop transmitter/receiver configuration^23^.

The Transient Electromagnetic (TDEM) method was applied to investigate subsurface resistivity variations, providing detailed information on groundwater-bearing formations. GPS coordinates of each loop center were recorded using a handheld Garmin device. Data were acquired at multiple voltage levels (0.1, 1, and 10 V) to capture different subsurface features. Voltage decay curves were averaged to reduce noise, and inversion was performed to produce resistivity-depth models using ZondTEM1D software^24^. Consistency between adjacent sites was checked to ensure geologically reasonable results.

### Magnetic survey

Magnetic data were collected using GSM-19 v7.0 magnetometers with a sensitivity of 1 nT. Measurements were taken at 335 stations along 14 profiles with a spacing of 20–50 m, while a fixed base station recorded data for diurnal correction. Each site was measured three times at five-minute intervals, and readings deviating by more than 5 nT from the mean were excluded. The data were corrected and reduced to the pole (RTP) and separated into regional and residual components using radially averaged power spectrum analysis to highlight both deep and shallow structures. Quantitative interpretation involved 2D forward modeling and 3D Euler deconvolution, utilizing a model grid of 250 × 250 × 100 m and depth weighting. Edge detection techniques, including the tilt derivative (TDR) and analytical signal (AS), were also applied to delineate contacts and structural features. All magnetic data processing and analysis were performed using Oasis Montaj™ software (v8.3)^25^.

### Hydrogeology and hydrochemistry

Hydrogeological studies were conducted to confirm the validity of the geophysical results and to detect the groundwater aquifer parameters. These studies include examining the lithological formations and analyzing the results of pumping tests for three wells drilled at depths ranging from 181 to 225 m (wells 4, 5, and 6). Groundwater quality was also examined by analyzing water samples from all six groundwater wells in the area (Fig. 1).

To determine the hydraulic parameters of the aquifer in the study area, the results of pumping tests were analyzed with the help of Environmental Simulation, Inc. AquiferWin32 ®^26^. The constant pumping test is performed to estimate the hydraulic parameters of transmissivity (T), hydraulic conductivity (K), and storage coefficient (S) through interpretation with a semi-log plot^27^. Groundwater monitoring and recording of water level changes in wells are conducted until the drawdown in the monitoring wells approaches a steady state. Transmissivity and storage coefficient were estimated using the following equations:

1 $$T=2.3Q/4\pi \Delta s$$

2 $$S=2.25T{t}_{^\circ }/{r}^{2}$$

where Δs is the drawdown difference per one log cycle of time, t_o_ is the value of t when the drawdown is equal to zero, and r is the distance between the observation and the pumping well.

Groundwater geochemistry was examined through the analysis of 6 groundwater samples collected from the pumping wells and analyzed at the central laboratory for water quality analysis at Sohag Holding Company for Water and Wastewater. The pH and total dissolved solids (TDS) have been measured in situ using pH and TDS meters, respectively. The concentrations of major cations (Ca^2+^, Mg^2+^, Na^+^, K^+^) were quantified in the laboratory using inductively coupled plasma mass spectrometry. The Cl^−^ and HCO_3_^−^ concentrations were measured by titrimetric methods, while SO_4_^2−^ and NO_3_^−^ were assessed using a spectrophotometer device.

## Results and discussion

### Magnetic results

The examination of magnetic data is essential for identifying subsurface magnetic features, particularly in the basement rocks. Qualitative interpretation provides a general overview of magnetic anomalies displayed on magnetic maps and the characteristics of their underlying sources. In contrast, quantitative interpretation employs modeling and inversion methods to determine the depth of the crystalline basement surface^28,29^. Sedimentary rocks that lack iron ore or basic intrusions are generally non-magnetic, whereas metamorphic and igneous rocks are highly magnetized^30^. Consequently, the thickness of sedimentary rocks can be inferred by estimating the depth of the magnetic sources, namely, the basement rocks^31^. To investigate the subsurface structure of the study area and to delineate features such as the active emergency spillway fault, multiple analytical techniques were applied to the magnetic grid data. These techniques included adjusting the data to a standard reference point, separating the overall and local magnetic signals using a specific method, applying filters, estimating depth, analyzing tectonic trends, and using both forward and inverse modeling.

### Qualitative interpretation of magnetic data

**Fig. 3:**
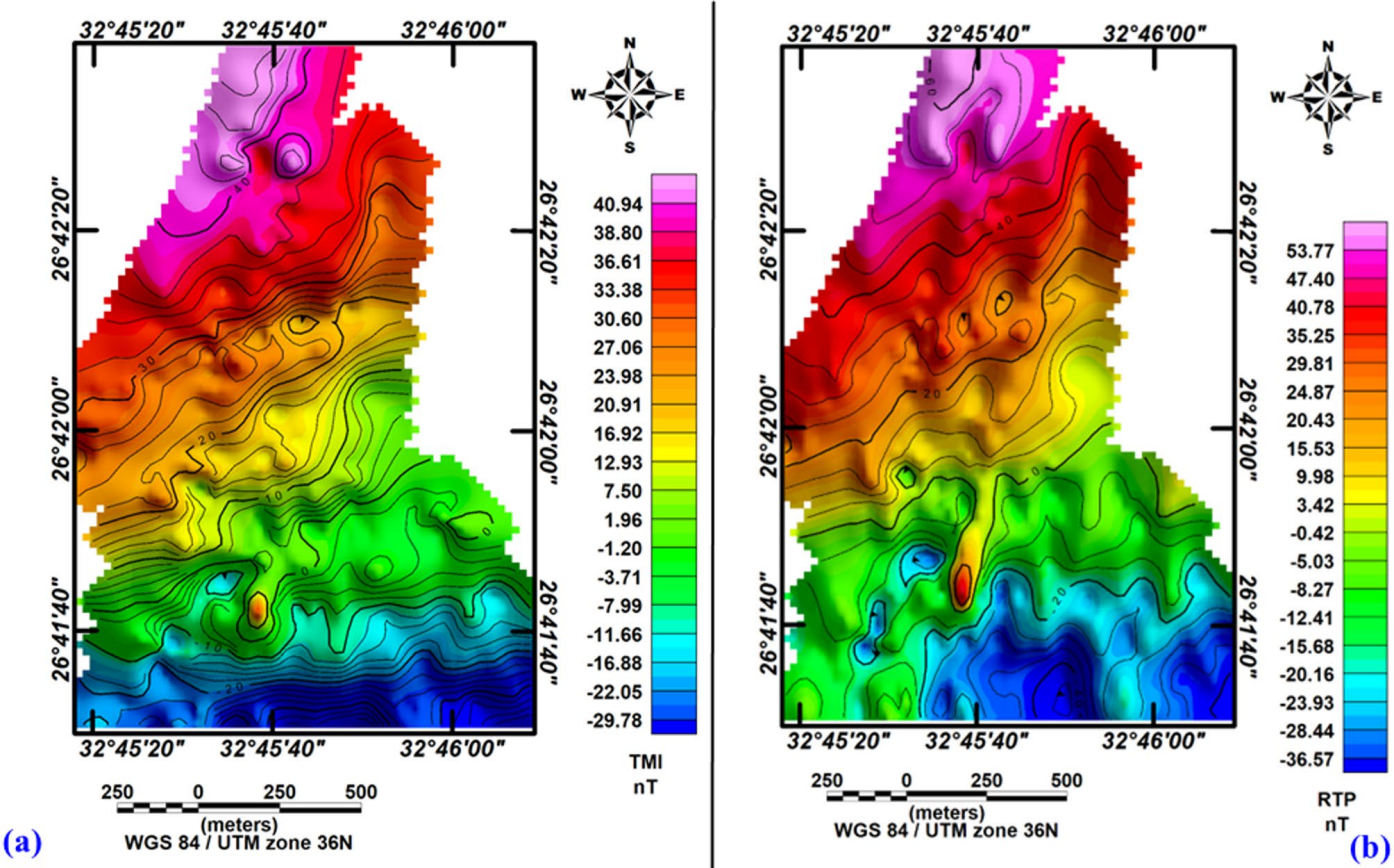
(**a**) Total magnetic intensity (TMI) map and (**b**) reduced-to-the-pole (RTP) magnetic map of the study area.

The total intensity magnetic map reveals a minimum value of − 30 nT and a maximum value of 50 nT, allowing for the classification of distinct zones based on their magnetic signatures (Fig. 3a). High magnetic anomalies, observed predominantly in the northern, northeastern, and northwestern regions of the study area, are characterized by positive values ranging from 25 to 50 nT. These high anomalies suggest the presence of a shallow basement, indicating a thin sedimentary cover in these regions.

In contrast, low magnetic anomalies, concentrated in the southwestern, southern, and southeastern portions of the study area, exhibit values ranging from − 8 nT to − 30 nT. These low anomalies are indicative of a deeper basement, corresponding to a thicker sedimentary cover. Additionally, areas with moderate magnetic anomalies, ranging from − 8 nT to 25 nT, exhibit various geometric shapes, including ovals, circles, and irregular forms.

The total magnetic intensity map is reduced to the pole using Oasis Montaj™ software (v8.3)^25^, with inclination (39°), declination (3°), and magnetic field strength (41595γ). The analysis of the resultant RTP map (Fig. 3b) indicates a minor discrepancy in the spatial positioning of the anomalies relative to the original data after adjustments were made. The map shows anomalies with magnitudes ranging from − 40 nT in the southern, southeastern, and western parts to 55 nT in the northern, northeastern, and northwestern parts of the area.

### Regional–residual separation

**Fig. 4:**
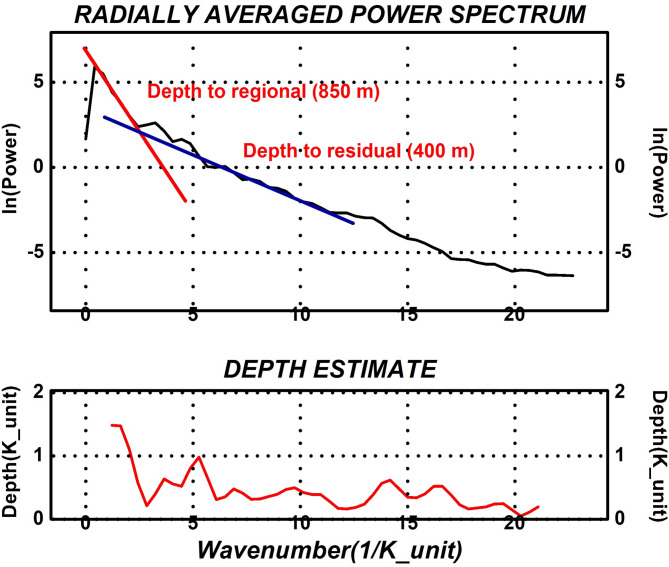
2D radially averaged power spectrum of the magnetic data.

In this study, Geosoft Oasis Montaj™ software (v8.3) was utilized to identify lineation resulting from structural faulting in basement rocks at varying depths, using 2-D filtering techniques applied to the RTP magnetic map. A cutoff frequency of 0.088 cycles per unit was selected, as it was suitable for the depth being analyzed on the aeromagnetic map (Fig. 4). Two types of filters were employed in the analysis: a residual (high-pass) filter and a regional (low-pass) filter. The low-pass filter allowed long wavelengths to pass through while rejecting those shorter than the cutoff wavelength. Conversely, the high-pass filter emphasized short wavelengths by eliminating those greater than the cutoff wavelength^32^.

**Fig. 5:**
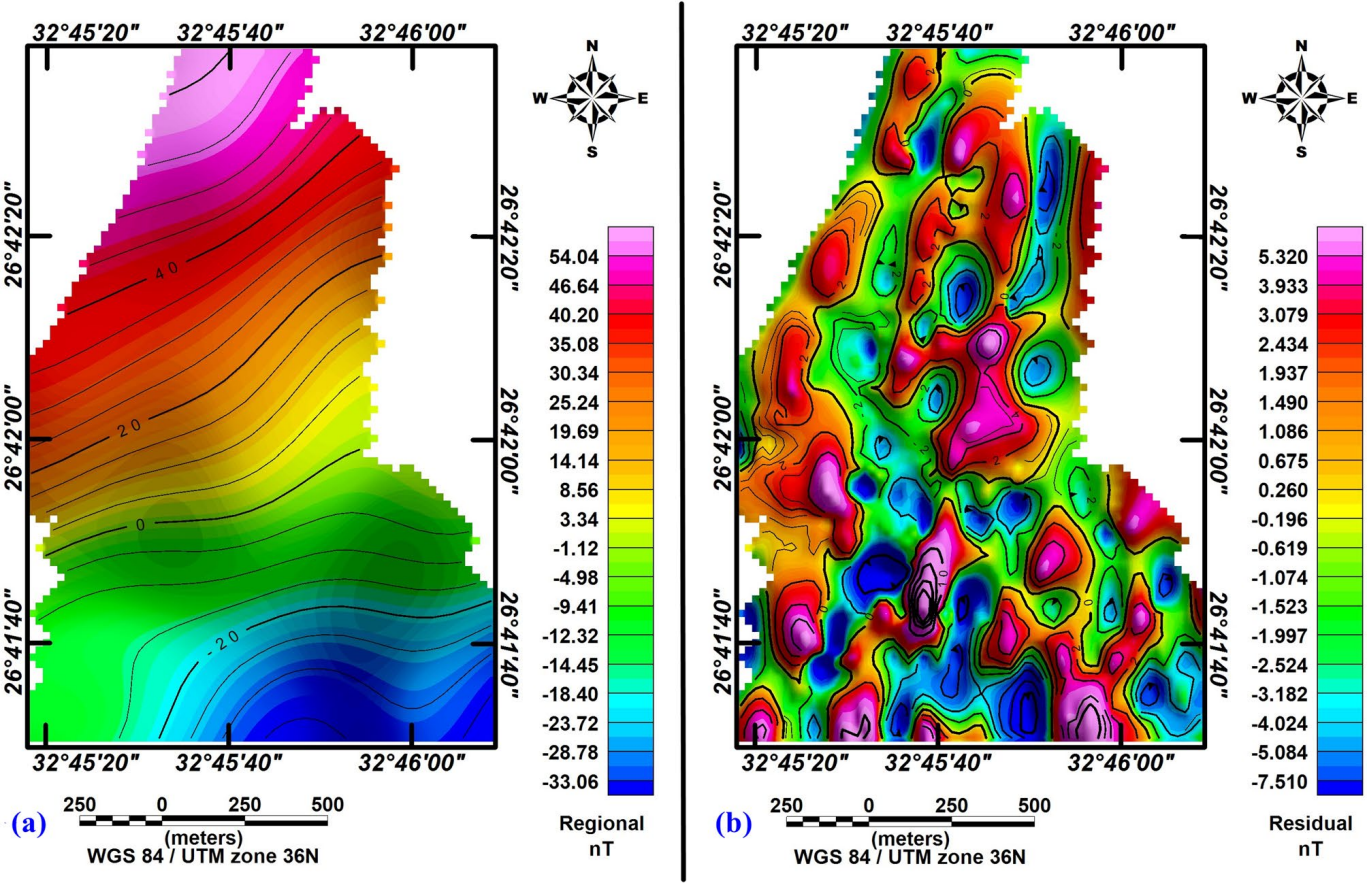
(**a**) Low-pass RTP map, and (**b**) high-pass RTP map for the investigated area.

The low-pass RTP magnetic filtered anomaly map (Fig. 5a) reveals that the well-defined trend of the RTP aeromagnetic map persists, reflecting the deep-seated subsurface structure responsible for the fault anomalies. The principal tectonic tendencies are controlled by deeper regional structures that trend NW–SE. Low magnetic anomalies located in the south with amplitudes of up to − 18.40 nT may be interpreted as structural lows or synclines. In contrast, high magnetic anomalies (high zones) cover the southern and southeastern parts, with amplitudes reaching 54.04 nT.

On the other hand, the high-pass RTP magnetic filter map provides insight into high-frequency and short-wavelength residual components present in various parts of the study area. The filtering process has resulted in numerous smaller anomalies with varying trends. However, the residual magnetic filtered map (Fig. 5b) shows that the patterns of the nearby structures are affected by the shallow local structures, which run north–south and northwest-southeast. These trends are associated with positive magnetic anomalies ranging from 0.2 nT to 5.3 nT and negative magnetic anomalies between − 7.5 nT and − 0.1 nT.

### Tilt derivative

**Fig. 6:**
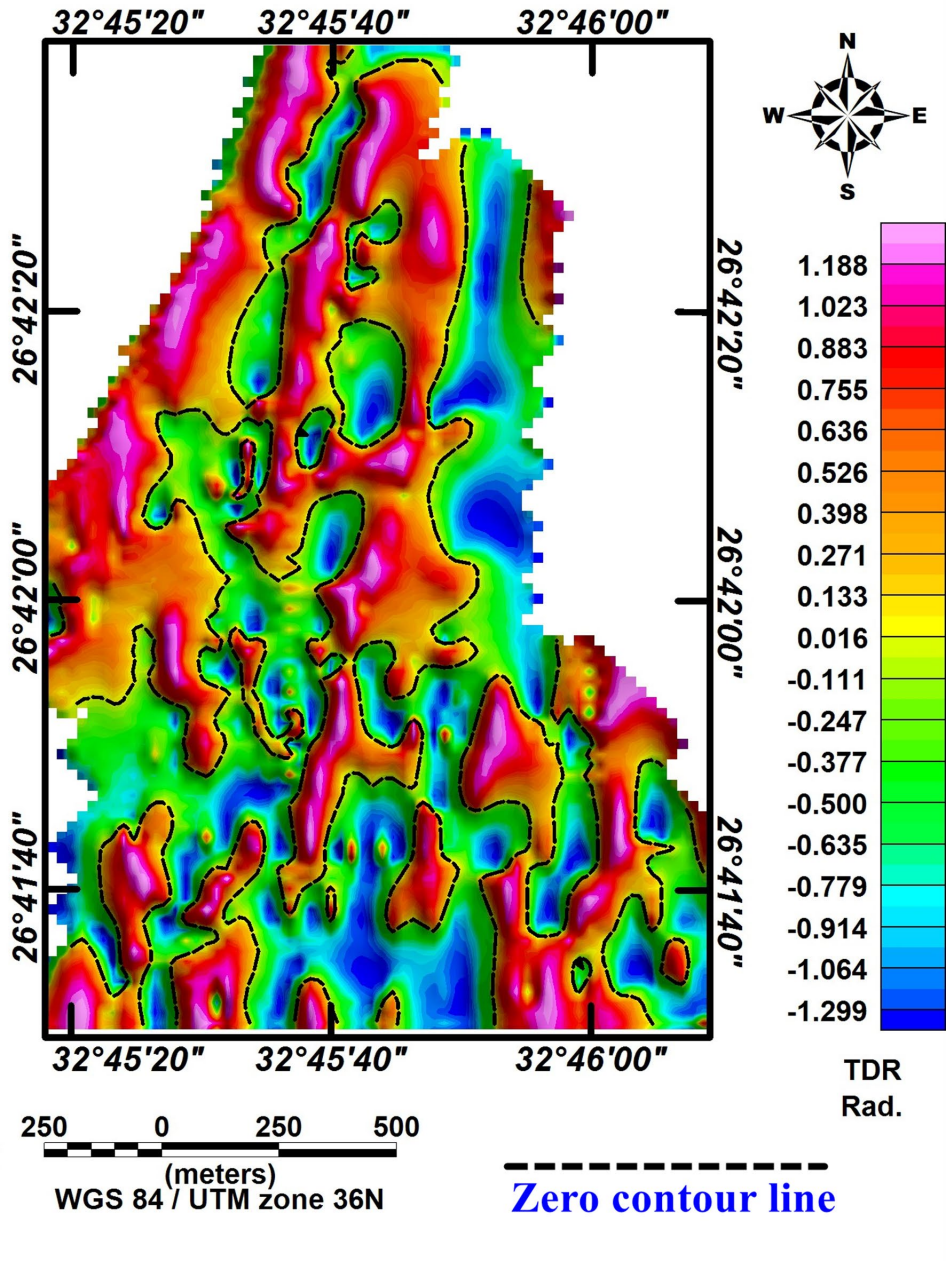
TDR of the RTP magnetic map of the study area.

The tilt derivative (TDR) is used to locate the horizontal position and extent of magnetic source edges, assuming a vertical contact model^33^. In the TDR map (Fig. 6), the zero-contour line (black line) marks the boundaries where sharp lateral changes in magnetic susceptibility occur, representing the edges of magnetic sources. Positive TDR values generally lie above the sources, while negative values are below them. The TDR map of the RTP magnetic data shows clear N–S and NW–SE lineament trends across the study area, reflecting the main structural directions.

### Quantitative interpretation for the selected profiles

**Fig. 7:**
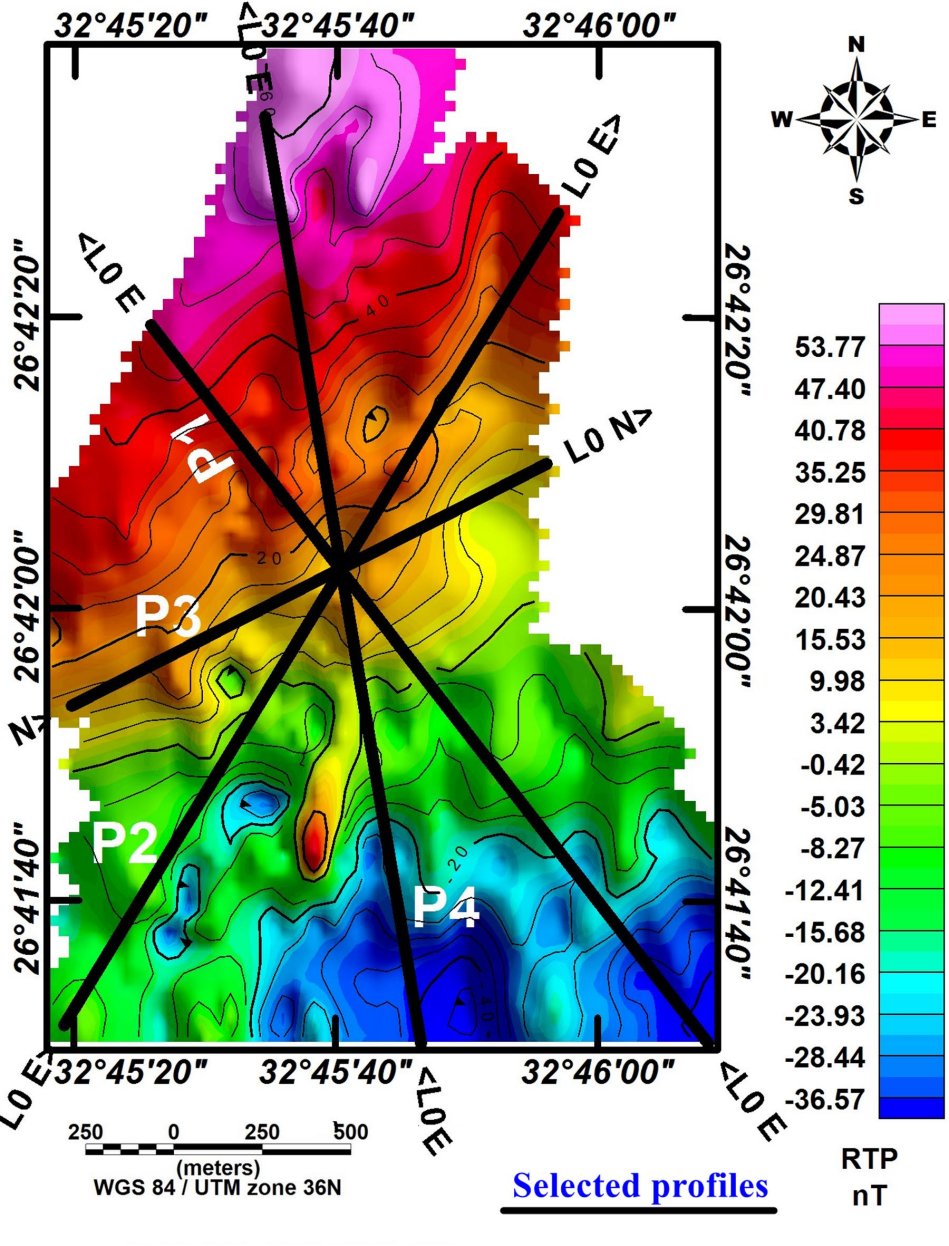
Map showing selected 2D profiles in the study area.

**Fig. 8:**
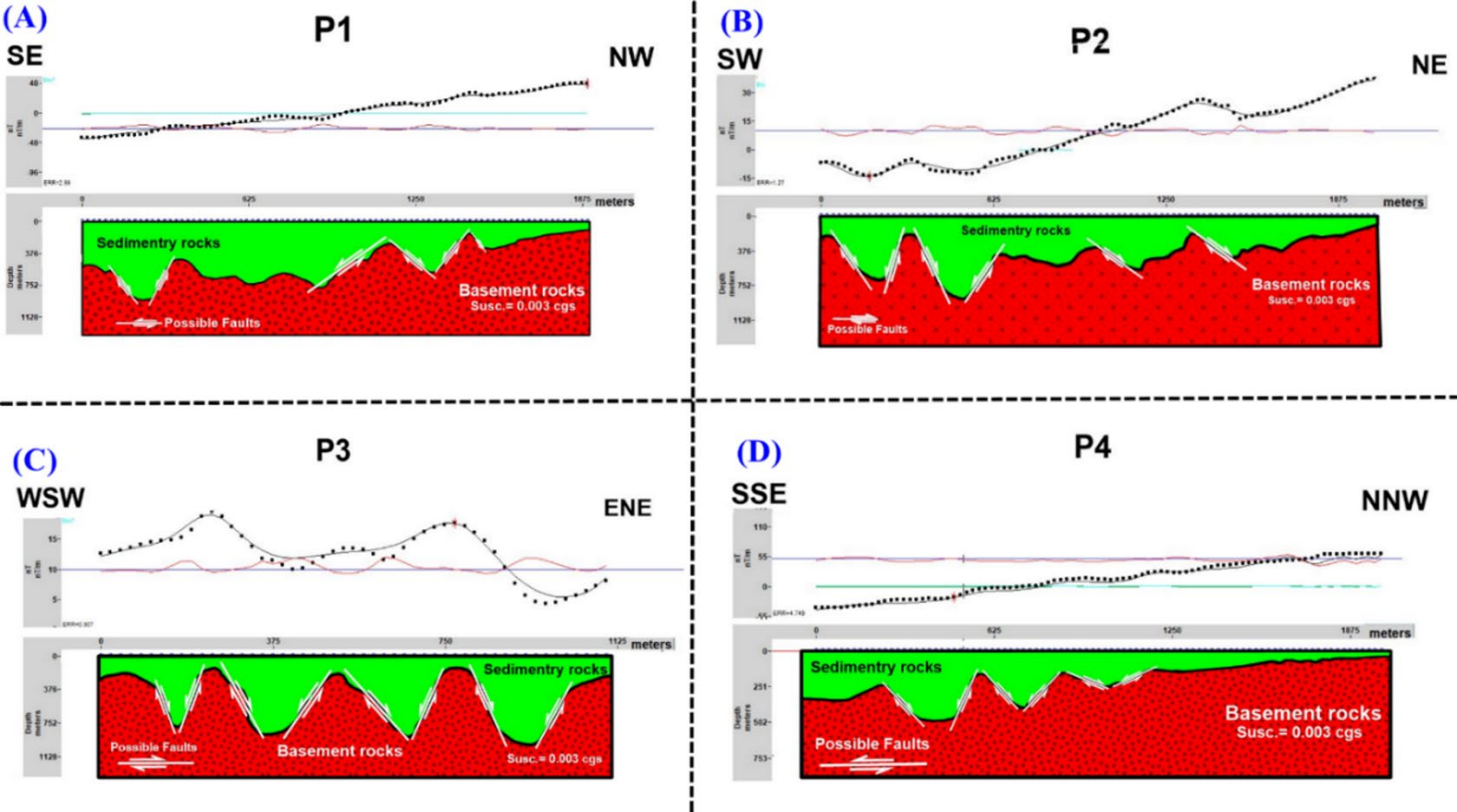
The inversion for four profiles (**a**–**d**, respectively) in the study area.

**Fig. 9:**
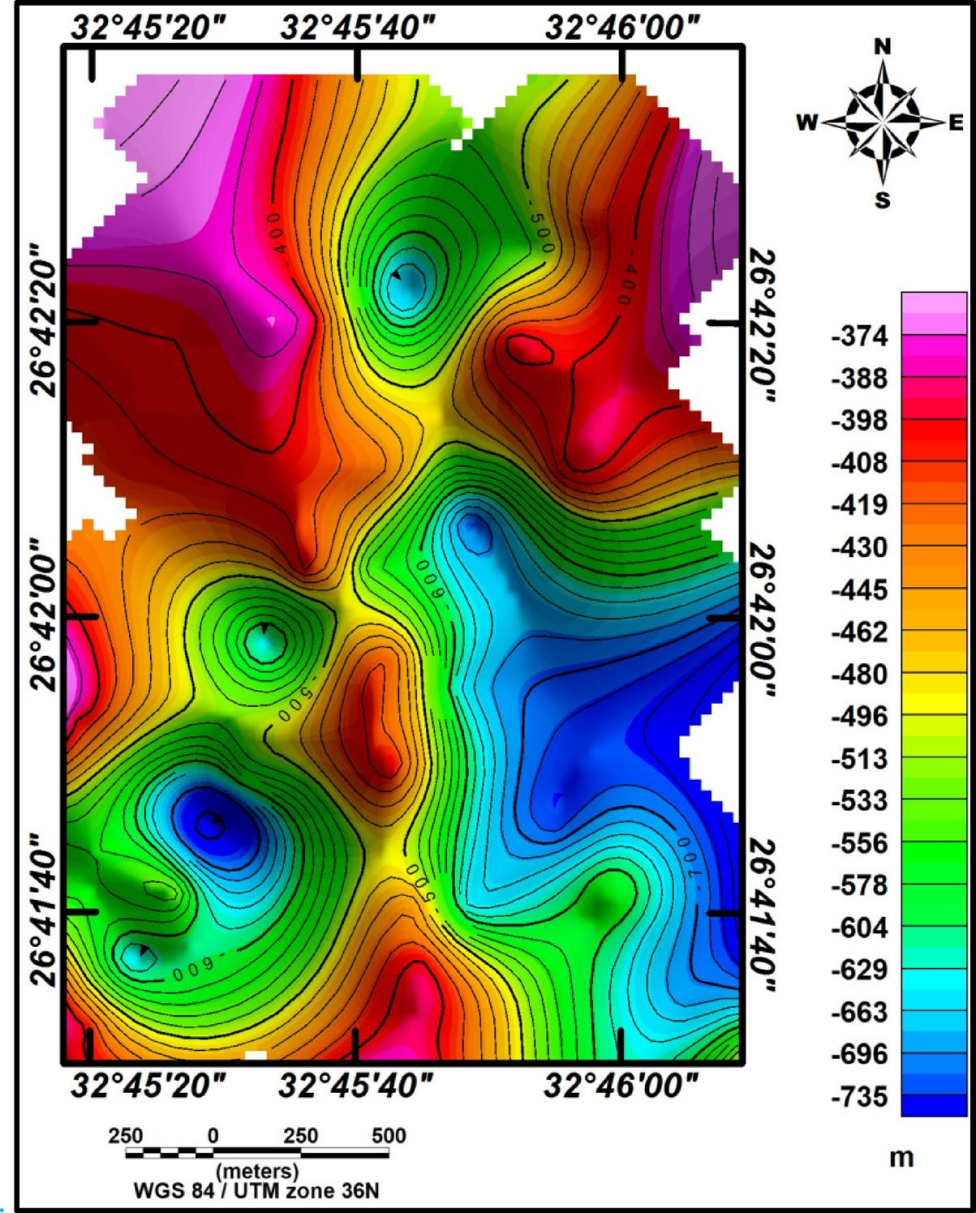
Basement relief map of the study area.

Four profiles were measured at chosen locations within the study area (Fig. 7). These profiles, labeled P1, P2, P3, and P4, were overlaid onto the RTP magnetic map, as shown in Fig. 7. The magnetic field for these profiles was computed iteratively until a satisfactory match between the observed data (dots) and the calculated data (line) was obtained. The magnetic interpretation based on 2-D models provided symmetrical findings across all profiles (Fig. 8a–d, respectively). Depth variations to the basement surface were observed, ranging from approximately 170 m to 800 m across shallow peaks. Fault/contact trends consistently aligned with NE-SW and NW–SE orientations, reflecting the broader geological trends observed in the Gulf of Suez and Gulf of Aqaba regions. The basement relief map illustrates significant depth differences across the study area (Fig. 9). Depths exceeding 600 m in the southeastern and southern parts indicate a thick sedimentary cover. In contrast, shallower depths below 400 m characterize the northern and northwestern regions. Trend analysis serves as an essential approach for comprehending the geometric aspects of geological and geophysical data across diverse categories.

### TDEM results

**Fig. 10:**
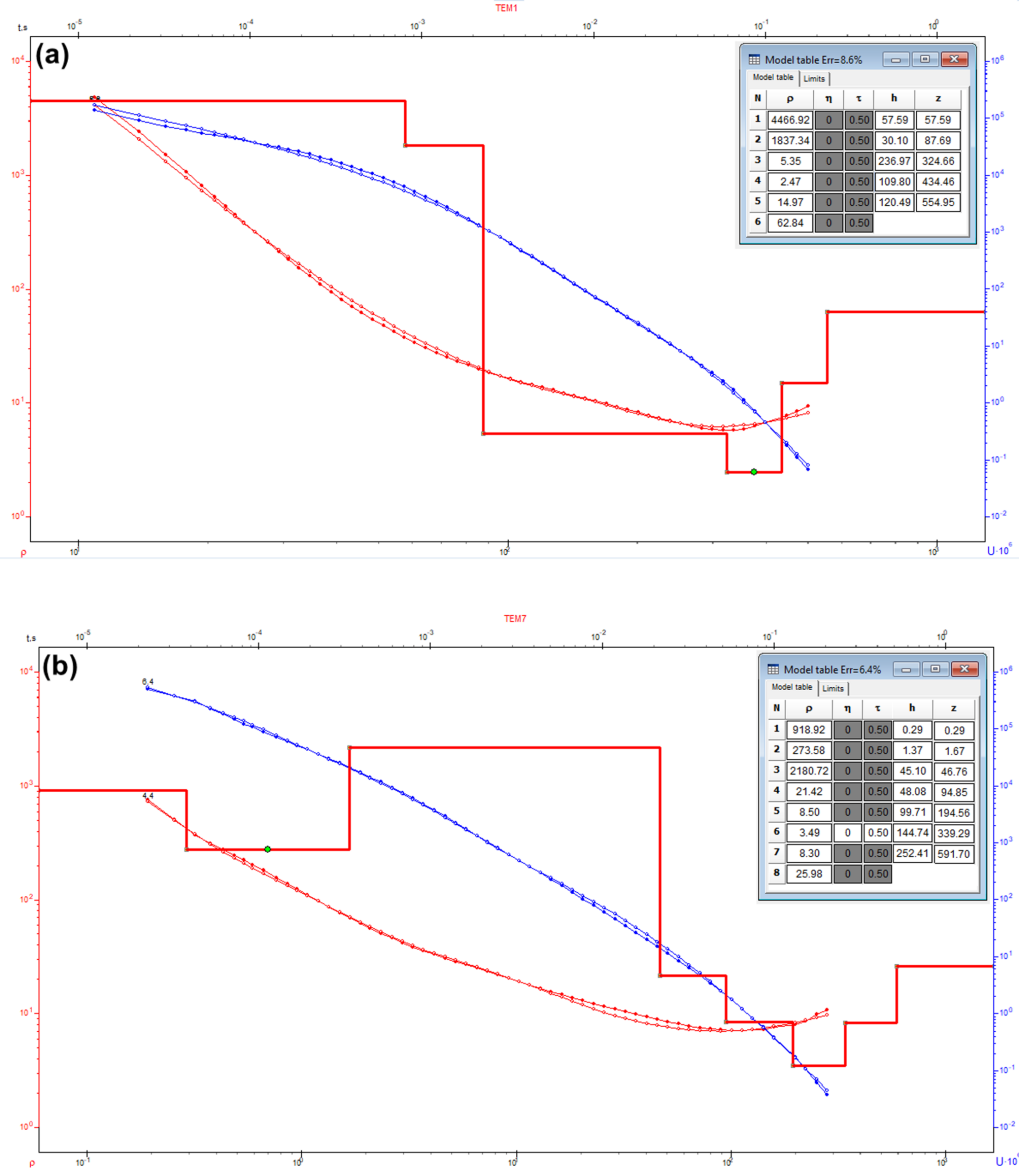
Example of TDEM sounding. Time-domain EM decay curves and 1-D layered inversion models for stations (**a**) TEM1 and (**b**) TEM7.

**Fig. 11:**
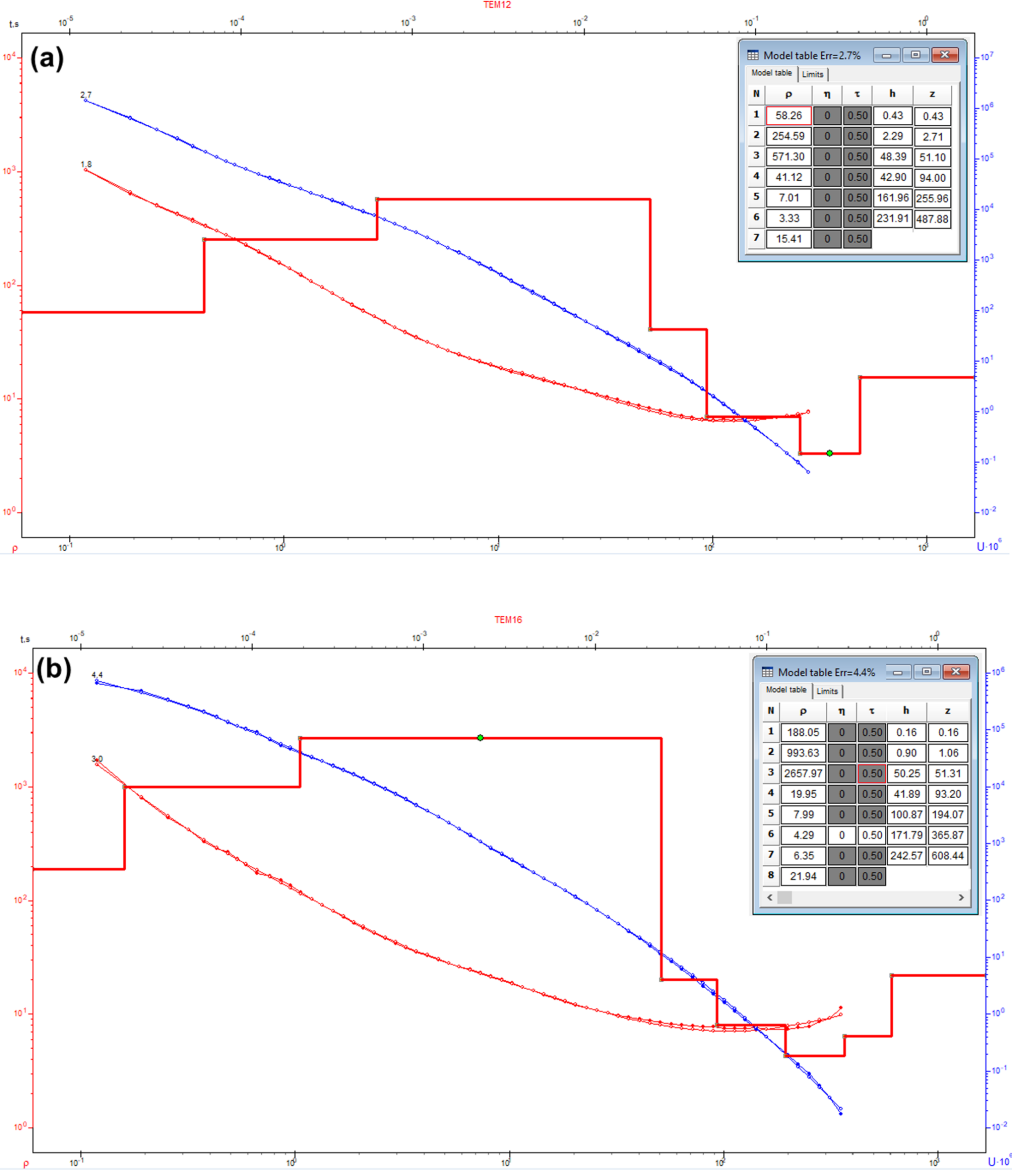
Example of TDEM sounding. Time-domain EM decay curves and 1-D layered inversion models for stations (**a**) TEM12 and (**b**) TEM13.

**Fig. 12:**
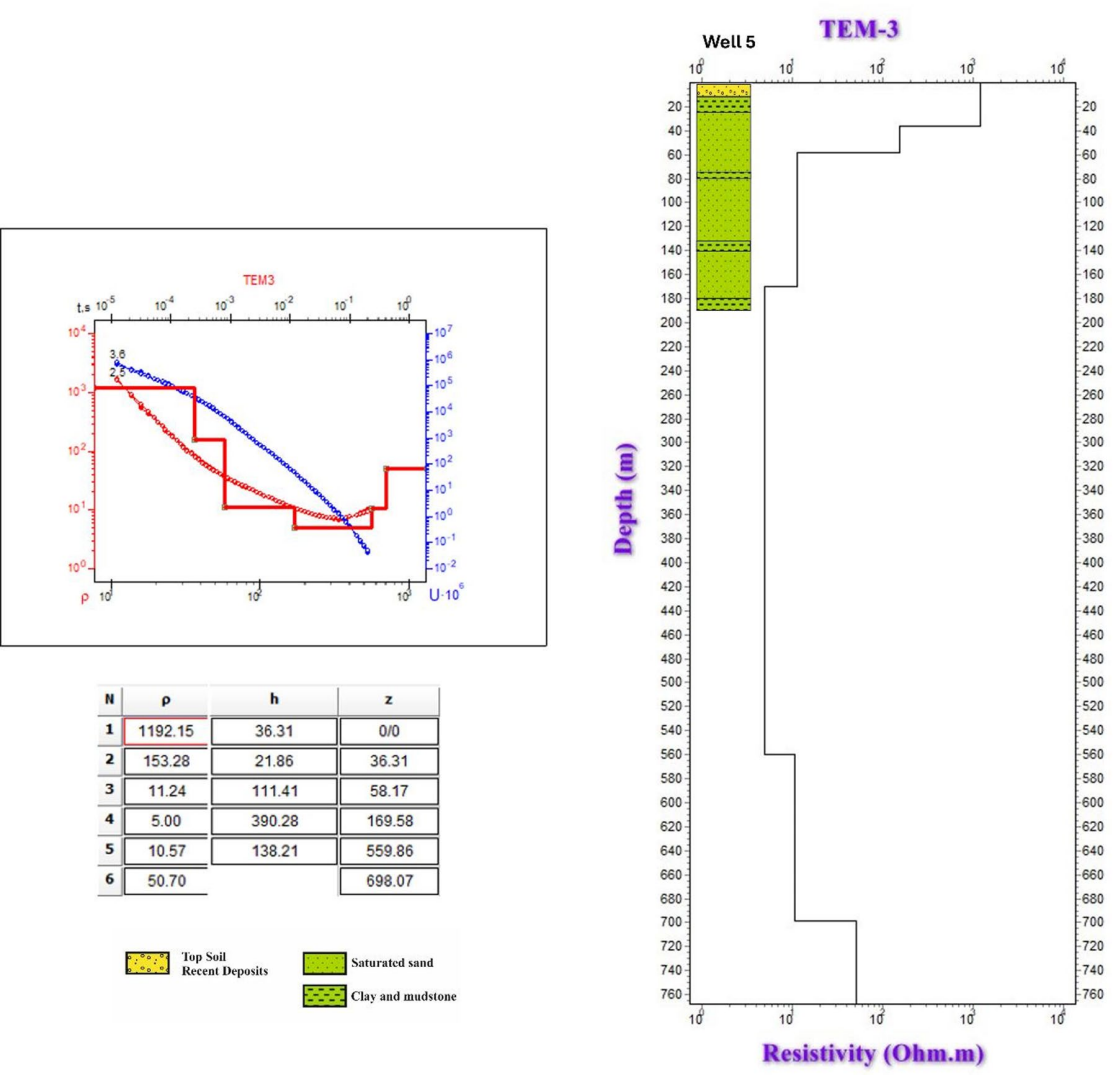
1-D Inversion of TEM-3 sounding data: apparent resistivity versus time curves and layered resistivity model validated against Well 5.

**Fig. 13:**
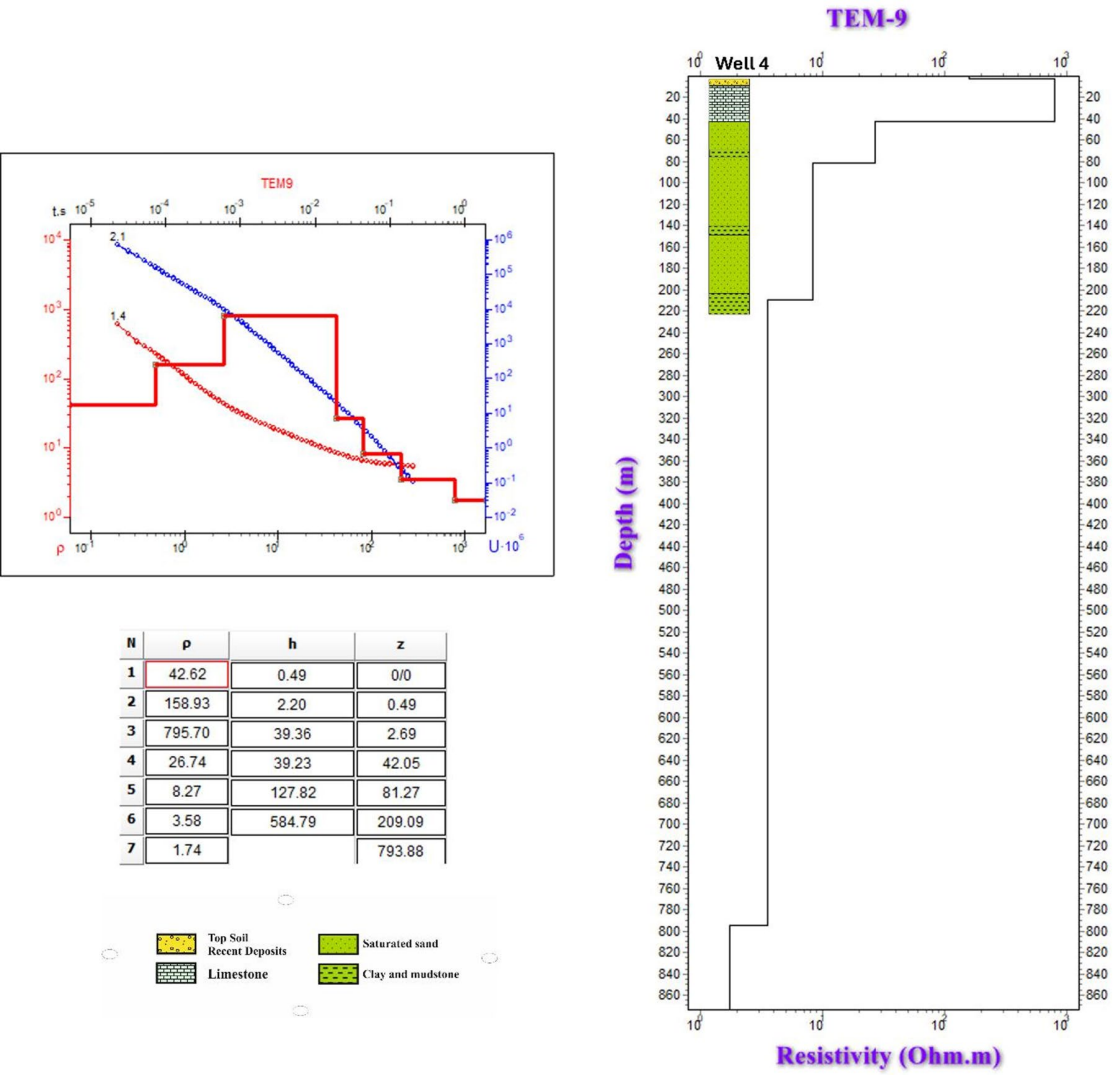
1-D Inversion of TEM-9 sounding data: apparent resistivity versus time curves and layered resistivity model validated against well 4.

**Fig. 14:**
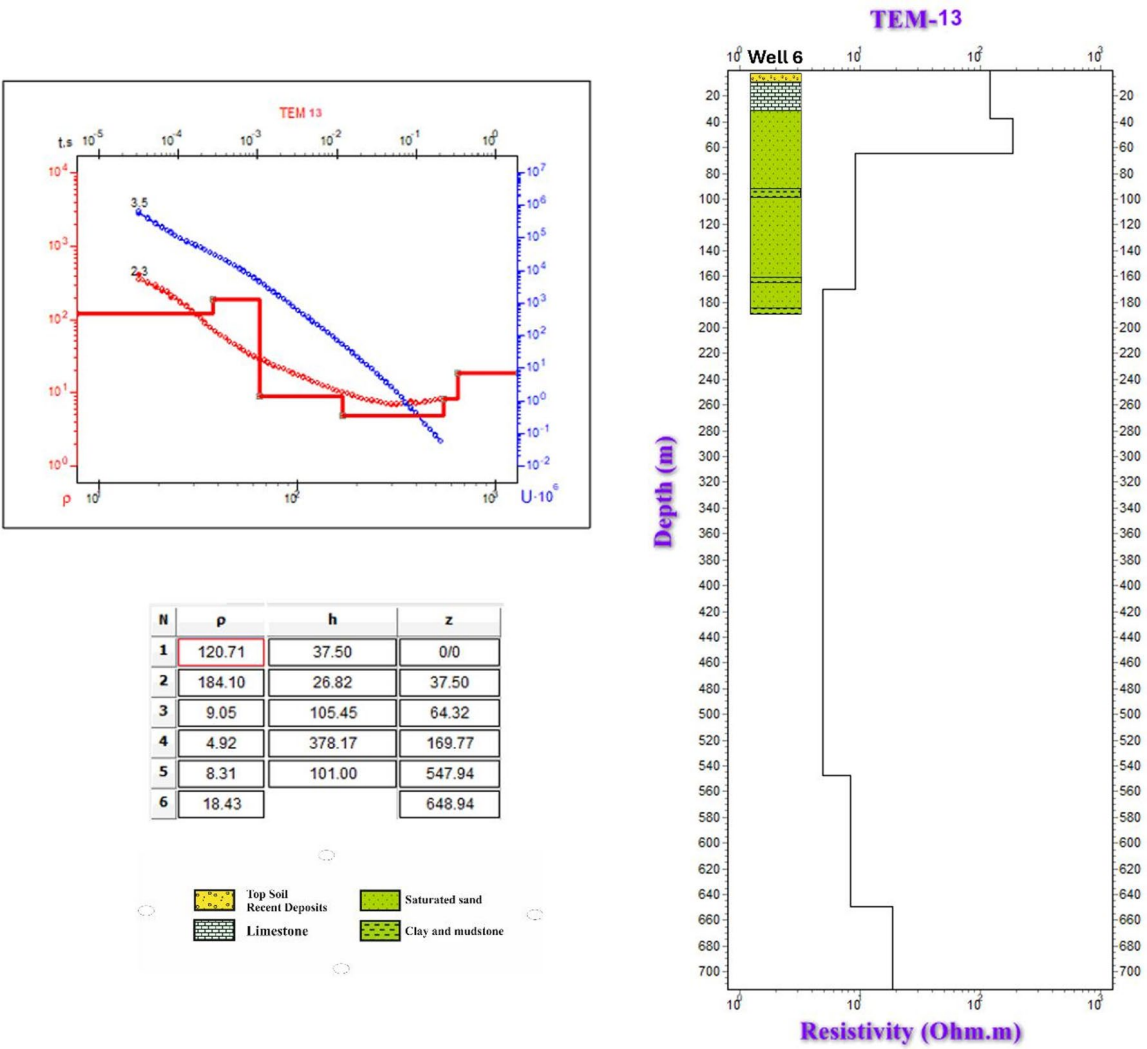
1-D Inversion of TEM-13 sounding data: apparent resistivity versus time curves and layered resistivity model validated against Well 6.

**Fig. 15:**
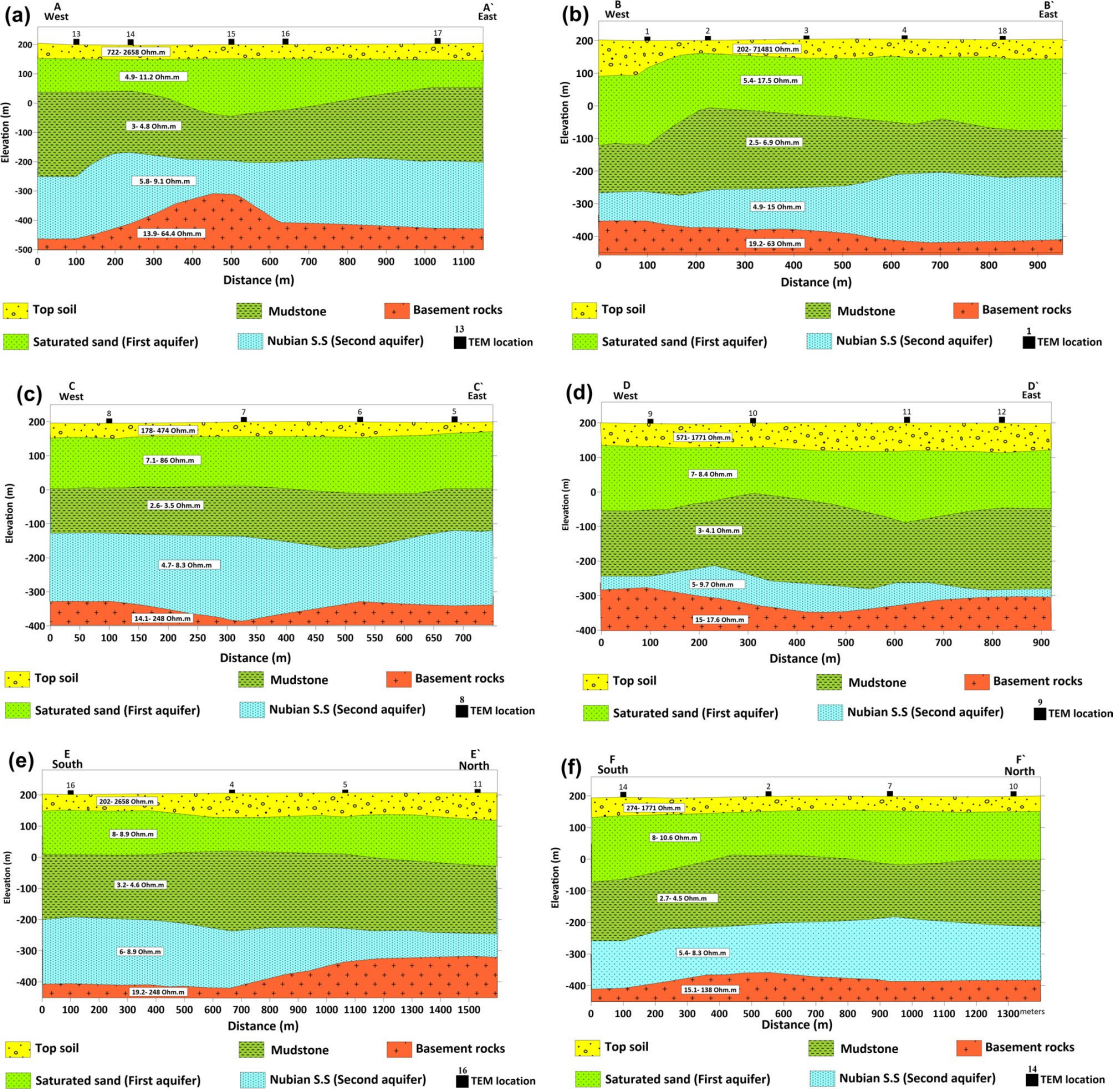
Hydro-geophysical resistivity model section for the study area for the study area and geoelectric cross-sections (**a**–**f**), produced by integrating 18 time-domain electromagnetic (TEM) soundings with the surface geological map and an interpreted geological cross-section. Resistivity (Ω m) and depth (m) scales are shown; interpreted lithologies and prospective water-bearing zones (annotated) are constrained by the combined TEM inversions and drilling data.

**Fig. 16:**
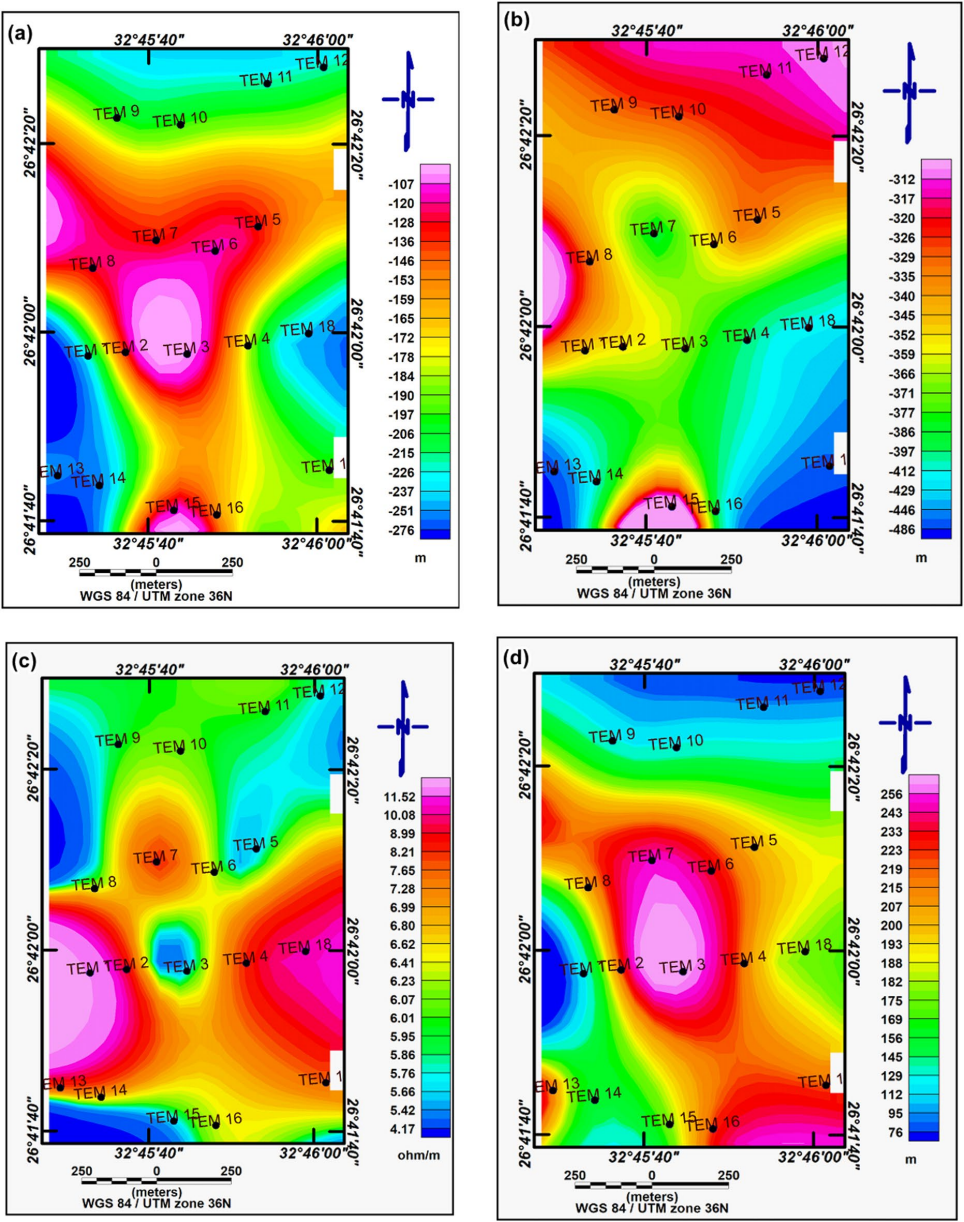
Spatial distributions for the Nubian Sandstone (main groundwater) aquifer: (**a**) depth to the top of the aquifer; (**b**) depth to the base of the aquifer; (**c**) aquifer resistivity; (**d**) aquifer thickness.

To assess the subsurface structure and delineate aquifer/aquitard geometry, transient electromagnetic (TEM) soundings were acquired and inverted using 1-D models (Fig. 1). Figures 10 and 11 show the observed and calculated TEM decay curves with 1-D resistivity models for stations TEM1, TEM7, TEM12, and TEM16, respectively. The models fit well (RMS 2.7–8.6%) and reveal resistive surface layers overlying conductive zones and deeper resistive units, indicating lithological or saturation variations. The 1-D inversion of the TDEM soundings was shown to correspond closely with the stratigraphy derived from drilling-sample intervals in nearby boreholes (Figs. 12, 13, 14). These correlations are considered to increase confidence that resistivity contrasts truly represent lithological transitions and changes in water saturation rather than artefacts. By integrating geophysical and borehole drilling sample data, the geometry, thickness, and resistivity of aquifer and aquitard units were delineated with greater certainty, thereby strengthening the hydrogeological model and refining the site-selection criteria for further drilling. To delineate the subsurface geo-electrical layers and identify the distribution of water-bearing zones, six comprehensive geo-electrical cross-sections were developed (Fig. 1). These sections extend across the study area, with four running from west to east (Fig. 15a–d) and two from north to south (Fig. 15e–f). Through the interpretation of Time Domain Electromagnetic (TDEM) data, a detailed subsurface profile emerged, revealing the presence of five different geoelectrical layers (Fig. 15a–f). These findings provide a clearer understanding of the subsurface composition and the distribution of aquifer formations within the study area. The first comprised Quaternary alluvial deposits of clay and sand, with average resistivity values ranging from 121.63 to 347,107.5 Ω-m, average thickness values ranging from 17.66 to 43.845 m, and average depth from 20.28 to 72.6 m. The second layer consists of Quaternary deposits, comprising sand and gravel, and is considered the shallow aquifer in the area. This layer exhibits a resistivity ranging from 5.35 to 17.5 Ω-m, with a depth varying from 113.42 to 324.7 m and a thickness ranging from 54.61 to 236.97 m. The third layer, composed of shale with marl, belongs to Upper Cretaceous deposits. This layer represents low resistivity values, ranging from 2.47 to 6.86 Ω-m, with a depth varying from 236.18 to 525.98 m and a thickness ranging from 97.47 to 331.2 m. The fourth layer, the Nubian Sandstone Formation, composed of sandstone with thin layers of clay belonging to upper Cretaceous deposits, represented the main groundwater aquifer in the region (Fig. 16a–d). This formation exhibits resistivity values ranging from 4.7 to 17.55 Ω-m, with depths varying from 501.53 to 667.14 m and thicknesses ranging from 120.49 to 325.07 m (Fig. 16a–d). The fifth layer, the basement rock, displays relatively high resistivity values ranging from 13.91 to 247.82 Ω-m. This layer, located below the Nubian Sandstone formation, has resistivity values typically exceeding 100 Ω-m, with depths ranging from 501.53 m. In the context of this study, the low resistivity of basement rocks typically indicates that these rocks are fractured and potentially filled with water or other conductive materials, such as minerals or brines. The main features of the Nubian Sandstone aquifer are resistivity values ranging from 4.17 to 11.52 Ω-m and thicknesses varying from 95 to 256 m. Northeastern, northwestern, and central regions have low resistivity values (less than 5 Ω-m), which, in contrast, indicates higher porosity and groundwater potential in those regions. Meanwhile, the southwestern and southeastern parts have high resistivity values of over 10 Ω-m. Thicker zones (over 200 m) are mainly in the central and southeastern sections, while thinner areas (below 150 m) are located in the northern, eastern, and western parts. These results are essential for effective groundwater management.

### Hydrogeological and hydrochemical properties

Three groundwater wells (wells 4, 5, and 6) were recently drilled in the study area to explore the subsurface lithology, verify the geophysical results, and conduct pumping tests through which the hydraulic parameters of the aquifer can be determined. The characteristics of these wells are described in Table 1. The lithology of the wells was compared to the geophysical data findings to confirm and verify the hydrogeological sequence of the area (Figs. 12, 13, 14, 15, 16).

**Table 1:** The characteristics and the resulting hydraulic parameters of the drilled wells in the study area.

Well no	Well depth (m)	Depth to water (m)	Static water level (m)	Well screens (m)	Transmissivity (m^2^/day)	Hydraulic conductivity (m/day)	Storage coefficient
4	225	63	147	90	1170.6	13	0.14
5	184	58	147	54	655.4	12.14	0.26
6	181	59	146	72	861.65	11.97	0.18

The lithological sequence of wells 4, 5, and 6 yields very consistent results with those of the spatially identical geophysical TDEM points 9, 3, and 13, respectively. The thickness of the top layer of the wadi deposits ranges between 13 and 15 m. It consists of rock fragments, sand, and clay intercalation, followed by dry fine to medium sand, clay, and limestone with a thickness ranging between 45 m in well 5 and 48 m in well 4 (Figs. 12, 13, and 14). Those two layers comprise the first geoelectrical layer identified through the TDEM survey. The first water-bearing layer was identified at depths of 63, 58, and 59 m below the surface, with thicknesses of 162, 126, and 122 m at wells 4, 5, and 6, respectively. This reflects the effective accuracy of the geophysical methods used.

**Fig. 17:**
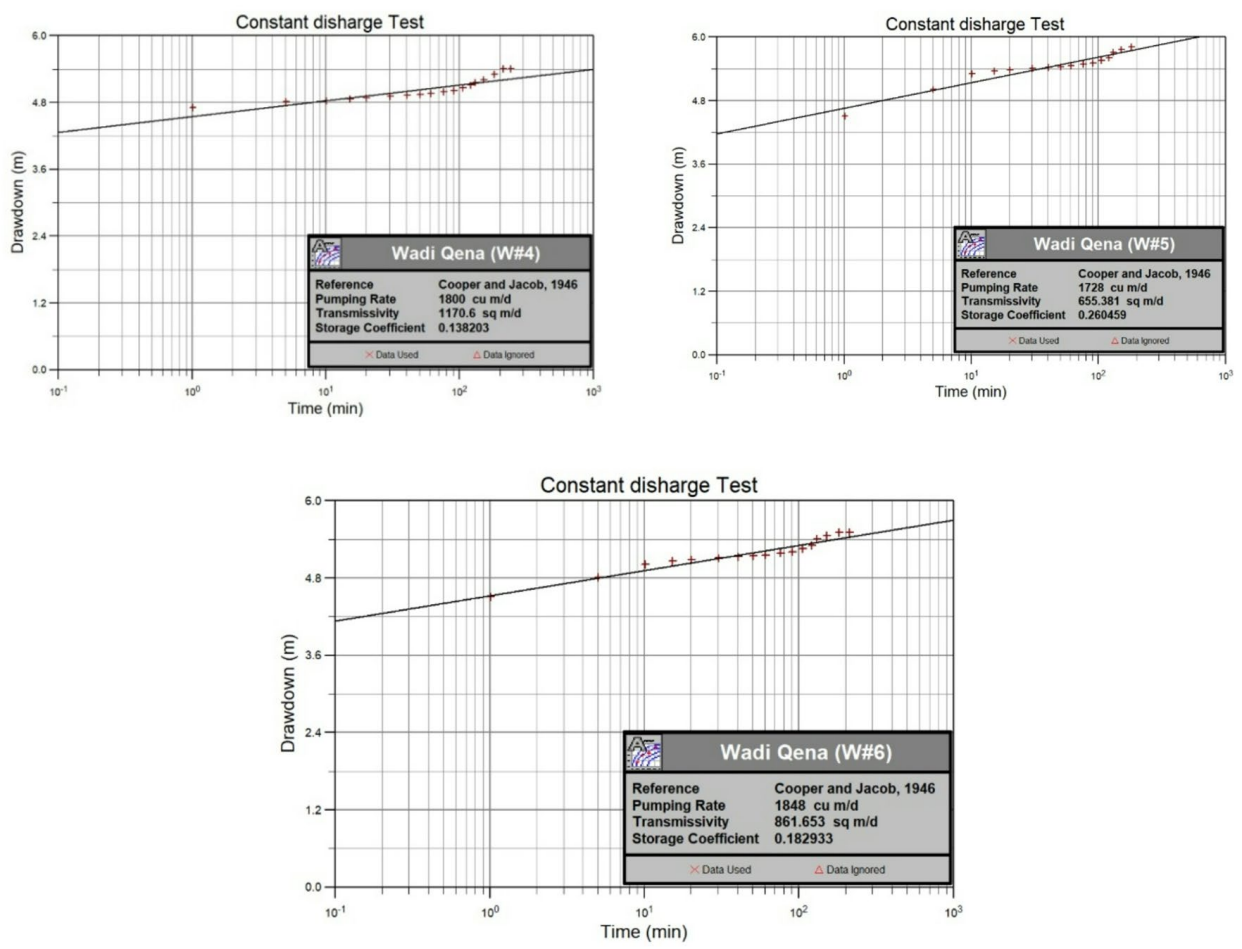
Pumping test analysis of the drilled wells in the area using the Cooper-Jacob method^35^.

The pumping test results of wells 4, 5, and 6 were analyzed and interpreted to determine the hydrogeological properties of the shallow (surficial) aquifer within the study area. The results of the pumping tests for these three wells are displayed in Fig. 17, and the calculated values for transmissivity (T), hydraulic conductivity (K), and storage coefficient (S) are listed in Table 1. The transmissivity ranges from 655 m^2^/day in well no. 5 to 1170.6 m^2^/day in well 4, reflecting a highly potential aquifer according to Gheorghe Standards^34^ Table 2. The high transmissivity in well 4 is due to increasing the thickness of the well penetration into the aquifer. The hydraulic conductivity values range from 11.97 m/day in well 6 to 13 m/day in well 4, with a mean value of 12.37 m/day, which corresponds to a coarse sand lithology, as indicated in Table 3. The storage coefficient value of the aquifer ranges from 0.14 to 0.26.

**Table 2:** Classification of the aquifer potential based on its transmissivity^34^.

Aquifer potentiality	Transmissivity
Highly potential	> 500
Moderate potential	500–50
Low potential	50–5
Very low potential	5–0.5
Negligible potential	< 0.5

**Table 3:** Hydraulic conductivity of different types of sediments^34^.

Type of material	Hydraulic conductivity (m/day)
Clay	10^–7^ to 10^–5^
Silt	0.1
Fine sand	0.1 to 1
Coarse sand	1 to 100
Gravel	100 to 1000 (or more)

**Fig. 18:**
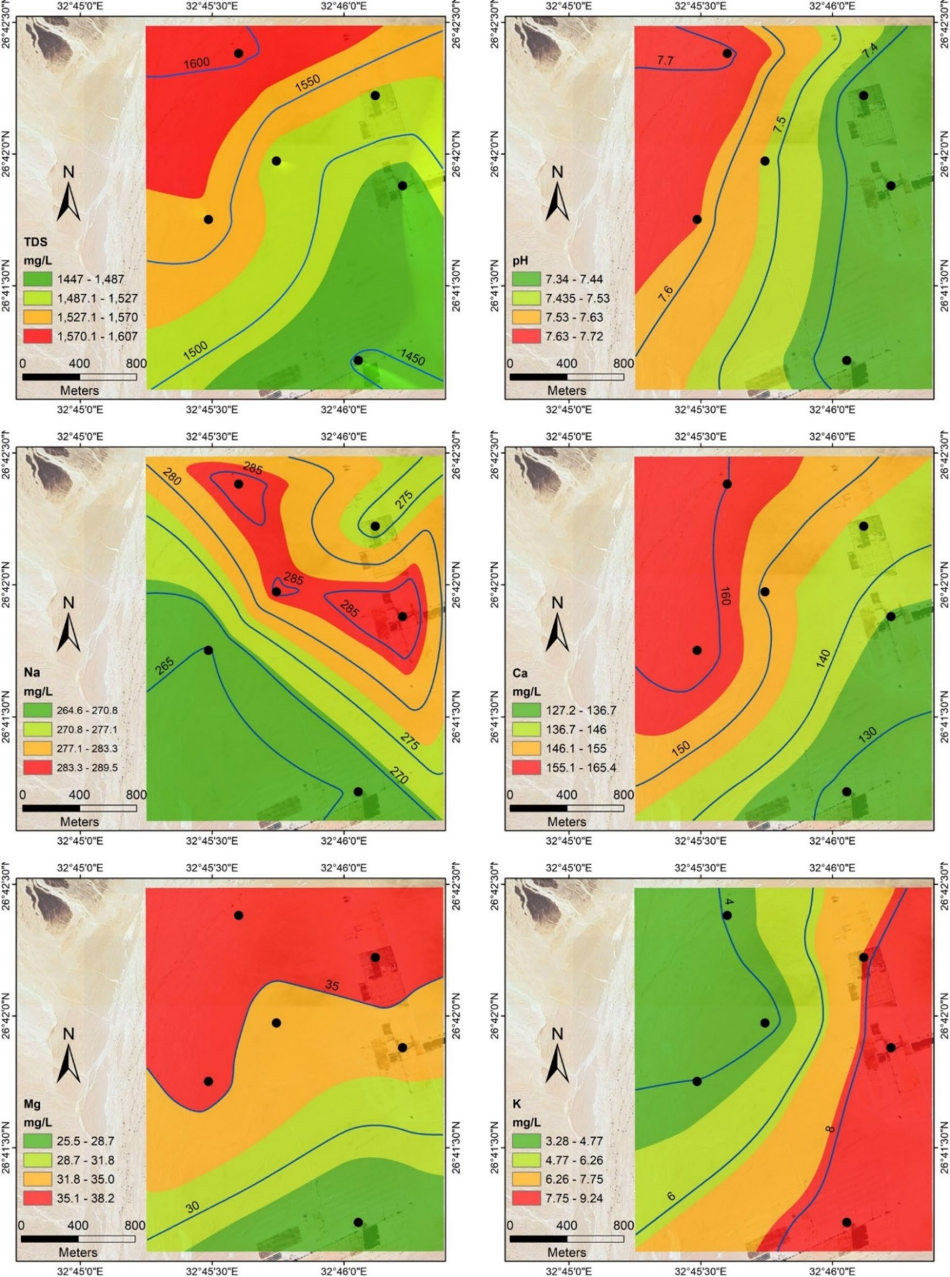
Spatial distribution maps of TDS, pH, and major cations (Na^+^, Ca^2+^, Mg^2+^, and K^+^) in groundwater samples from the study area.

**Fig. 19:**
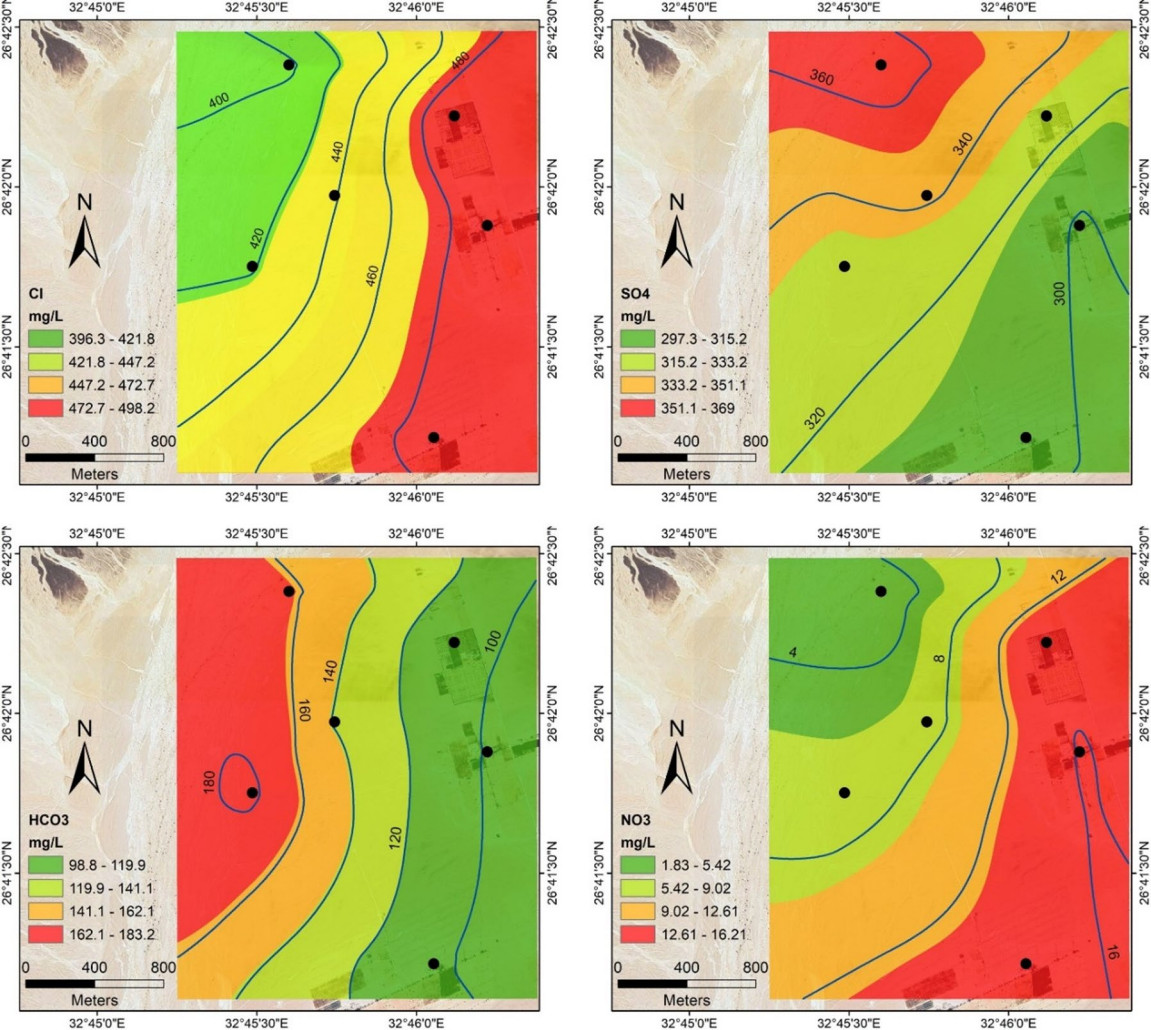
Spatial distribution maps of the major anions (Cl^-^, SO_4_^2-^, HCO_3_^-^, and NO_3_^-^) in groundwater samples from the study area.

Six groundwater wells have been sampled to study the geochemistry, quality, and suitability of groundwater for drinking and irrigation. The statistics for the measured physicochemical parameters of these samples are shown in Table 4. Total dissolved solids (TDS) vary from 1447 ppm in well 1 to 1607 ppm in well 4, with an average of 1521.33 ppm, increasing in the northwest direction towards the limestone plateau reflecting the leaching process of subsurface carbonate rocks intercalated with sand near to the plateau (Fig. 18). Ca^2+^, Mg^2+^, HCO_3_^−^, and SO_4_^2−^ concentrations have similar trends as TDS, increasing in the north and northwest direction, indicating natural activities and groundwater-rock interaction (Figs. 18, 19). In contrast, NO_3_^-^ and K^+^ concentrations increase in the south and southeast direction towards the agricultural areas where fertilizers and pesticides were used. Na^+^ and Cl^−^ spatial distribution maps indicating dominance and increase of sodium (Na^+^) and chloride (Cl^−^) concentration in the six investigated wells (Figs. 18, 19), indicating the sodium-chloride water type due to dissolution of halite and gypsum salts in groundwater, as well as the upward leakage from the Nubian Sandstone aquifer.

**Table 4:** The statistics for the measured physicochemical parameters are determined in the study area.

Parameter	Minimum	Maximum	Average	SD	Well of the highest conc	Well of lowest conc	WHO (2011)	Unsuitable samples
pH	7.34	7.72	7.49	0.15	Well 2	Well 1	8.5	None
TDS	1447	1607	1521.23	53.68	Well 4	Well 1	1000	100
Na^+^	264.57	289.5	277.52	10.37	Well 2	Well 6	200	100
Ca^2+^	127.2	165.4	146.95	13.08	Well 6	Well 1	75	100
Mg^2+^	25.5	38.2	33.90	4.33	Well 4	Well 1	35	3,4, 6
K^+^	3.28	9.24	6.211	2.46	Well 2	Well 5	12	None
Cl^-^	396.3	498.22	455.14	39.37	Well 3	Well 4	250	All
SO_4_^2−^	297.3	369	328.97	23.35	Well 4	Well 2	250	All
HCO_3_^-^	98.75	183.2	131.35	33.21	Well 6	Well 2	500	None
NO_3_	1.83	16.21	10.27	5.58	Well 2	Well 4	45	None

**Fig. 20:**
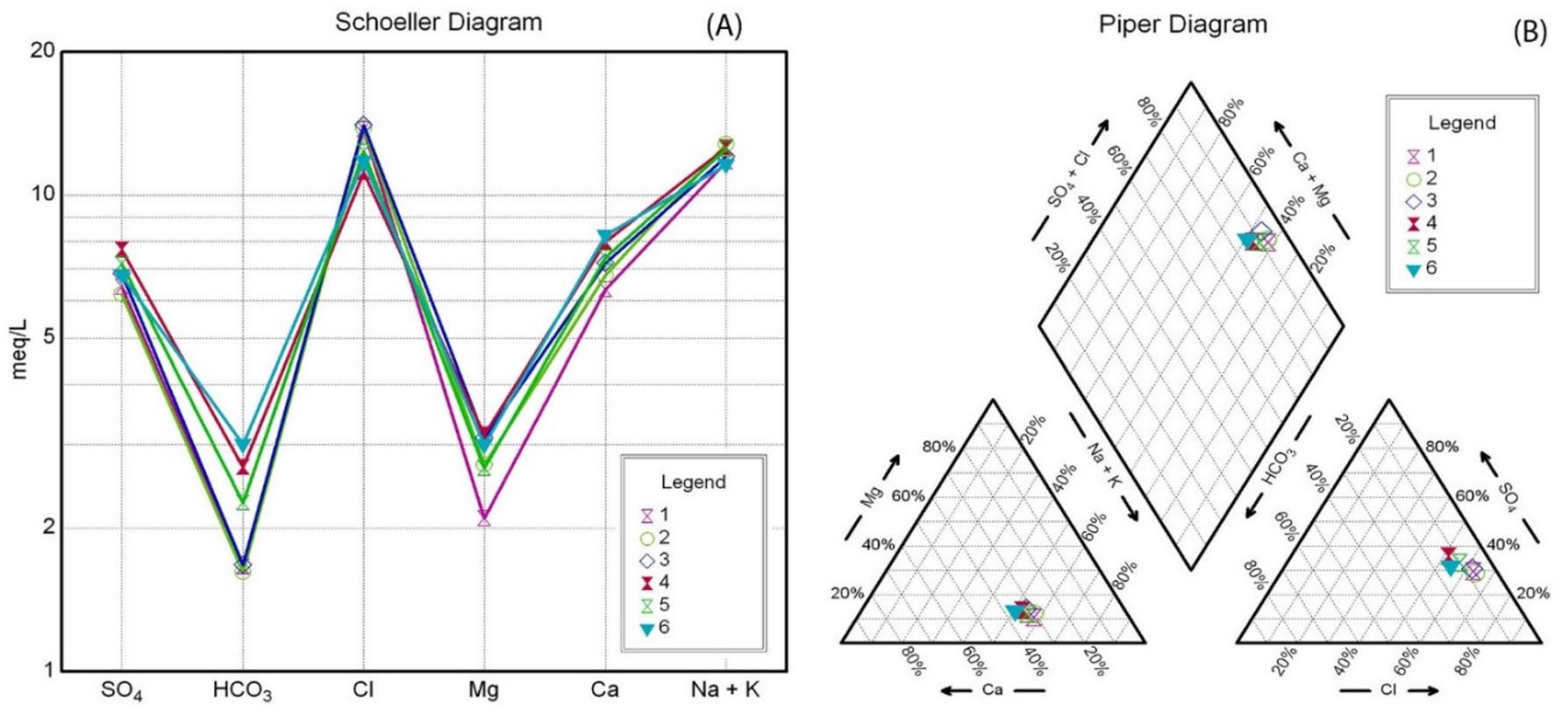
Graphical representation of Groundwater type; (**a**) Scholler Diagram^36^, and (**b**) Trilinear Piper diagram^37^.

The groundwater type in the study area was determined using the Scholler Diagram^36^, and Piper Diagram^37^. Scholler diagram (Fig. 20a) indicated that the abundance of major cations in the groundwater samples was ranked as: Na^+^  > Ca^2+^  > Mg^2+^  > K^+^, while major anion concentrations in the groundwater samples were ranked as: Cl^-^ > SO_4_^2-^ > HCO_3_^-^ > NO_3_^-^.

Moreover, it is shown that there is a similar approach to lines for all samples, indicating that the levels of Na + and Cl- are increasing and becoming more dominant in all the groundwater wells. This suggests that the groundwater is of a sodium chloride-water type, resulting from the dissolution of salts like halite and gypsum, as well as the mixing of upward-leaking groundwater with surface water that seeps in due to seasonal rains and floods in the area.

The Piper diagram (Fig. 20b) indicates that the groundwater in the study region revealed an alkali concentration (Na^+^  + K^+^) surpassing the alkaline earth concentrations (Ca^2+^  + Mg^2+^), and a strong acid content (Cl^-^ + SO_4_^2-^) exceeding the weak acid content (CO_3_^2-^ + HCO_3_^-^). This signifies that the groundwater is characterized by non-carbonated alkalis, and all samples from the studied wells are predominantly composed of sodium and chloride, due to the upward seepage of deep groundwater from the Nubian sandstone aquifer.

The measured parameters in groundwater samples have been compared with the World Health Organization guidelines for standard drinking water quality^38^. All collected samples exceeded the permissible limits according to WHO guidelines for Na^+^, Ca^2+^, Cl^-^, and SO_4_^2-^ concentrations, while they were acceptable for NO_3_^-^ and HCO_3_^-^ concentrations. However, they were unacceptable in samples 3, 4, and 6 and acceptable in the others for Mg^2+^ concentrations. TDS values for all samples are more than 1000 mg/L, reflecting their unsuitability for drinking and other domestic uses. However, the water quality falls within the range of brackish water and may be suitable for irrigating specific crops that can tolerate elevated levels of salinity.

**Fig. 21:**
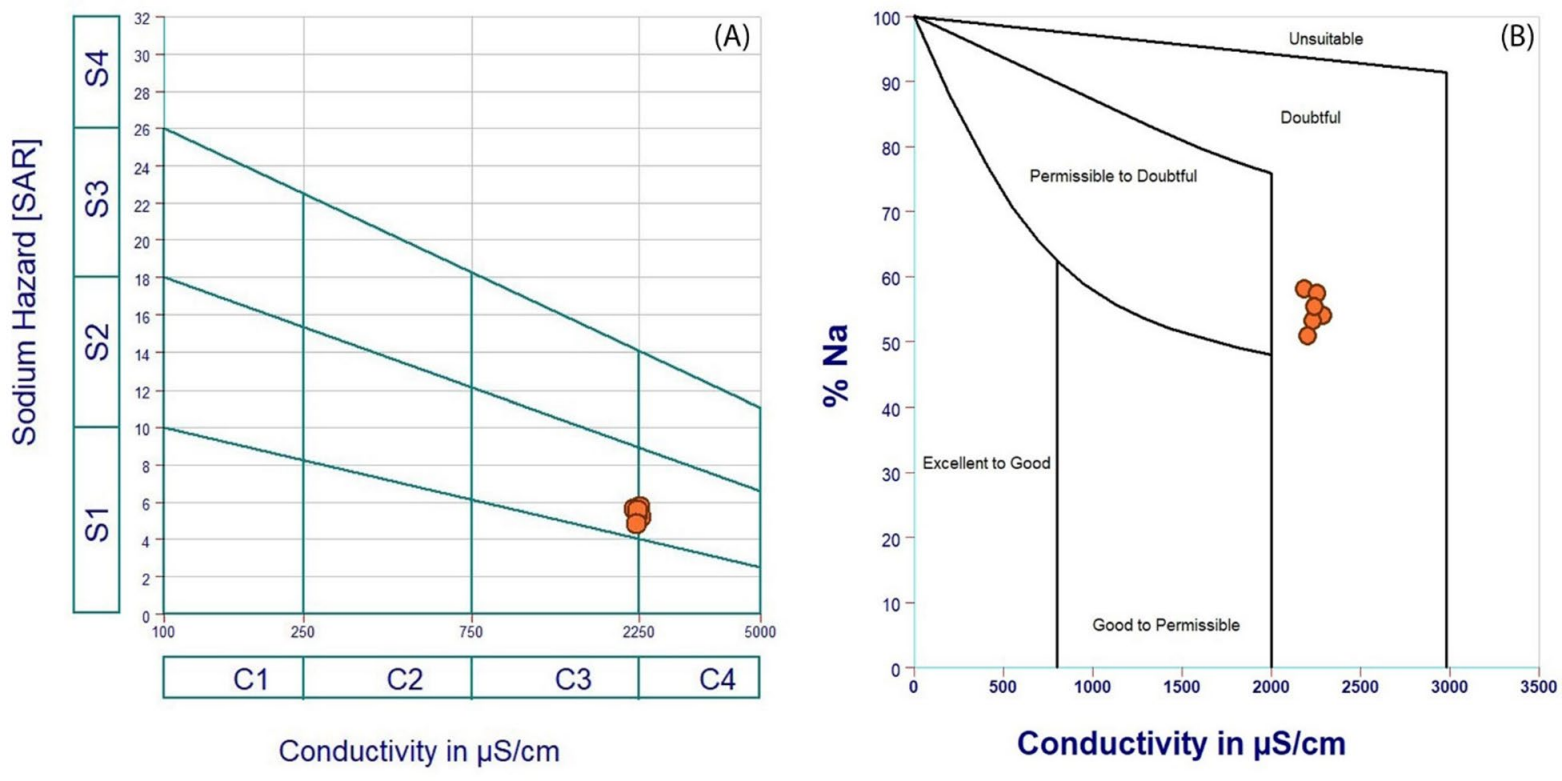
Groundwater suitability for irrigation according to (**a**) the US Salinity Laboratory^39^, and (**b**) Wilcox diagram^40^.

Figure 21a illustrates the plotted values of SAR against EC, as proposed by the United States Salinity Laboratory^39^. This diagram indicates that all groundwater samples fall into the “moderate” category for irrigation (group C3–S2), in which it can be used for irrigation but requires good drainage and crop rotation with salt-tolerant crops. The Wilcox diagram^40^ illustrates the quality of irrigation water by plotting Na% against EC (Fig. 21b). The findings indicated that all groundwater samples were categorized as “doubtful.” Consequently, groundwater may be utilized for irrigation purposes under specific conditions.

## Conclusion

A promising area for sustainable development in Wadi Qena has been studied using an integrated approach that combines geophysical, hydrogeological, and hydrogeochemical methods to evaluate groundwater potential and subsurface characteristics. Advanced techniques such as Time Domain Electromagnetic (TDEM) and magnetic surveys were used to classify and analyze the main aquifer systems, providing valuable insights into the region’s groundwater resources. The Quaternary shallow aquifer, located at depths of approximately 50–300 m with resistivity values ranging from 4.9 to 86 Ω-m, is identified as a secondary aquifer with limited capacity. In contrast, the Nubian sandstone aquifer, located at depths ranging from 300 to 650 m with resistivity values between 4.7 and 17.55 Ω-m, is a significant primary aquifer essential for the regional water supply. Magnetic surveys reveal basement depth variability ranging from 350 m to over 850 m, with a thicker sedimentary cover in the southern and southeastern areas, suggesting a higher groundwater storage potential. The northern areas show shallower sediment thickness, indicating lower storage potential. High transmissivity values, up to 1170.6 m^2^/day, in the shallow Quaternary aquifer indicate excellent groundwater potential for extraction. At the same time, hydraulic conductivity ranges from 11.97 to 13 m/day, reflecting the homogeneity of the sedimentary medium, which consists of coarse sand formations. Hydrogeochemical analysis reveals higher levels of Total Dissolved Solids (TDS), ranging from 1447 to 1607 ppm, and elevated ion concentrations, indicating water quality issues that limit its suitability for domestic use. Some areas are classified as brackish and are primarily suitable for irrigation under controlled conditions. This approach, combining geophysical exploration with validation through drilling new wells in desert areas, will open new horizons for sustainable development in these regions. More efforts should be made in these areas to drill deep wells that extract water from high-potential aquifers, ensuring the qualitative and quantitative sustainability of water.

## Data Availability

All datasets generated and/or analysed during this study were collected for the first time through this work and are not registered in any repositories or taken from any organizations or authorities. The data that support the findings of this study are available from the corresponding author upon reasonable request.
